# “What I wish I had known before starting my PhD”

**DOI:** 10.1002/ansa.202200044

**Published:** 2022-11-30

**Authors:** Vincent Wiebach

**Affiliations:** ^1^ Department of Biotechnology and Biomedicine Technical University of Denmark Lyngby Denmark

## Abstract

As a rather recent PhD graduate and still an “early career researcher”, the author wondered what to write about that would be interesting for a young scientist. The answer came while overhearing students in the break room stating, “I wish I had known all that before starting my PhD that would have made everything easier!” – An experience many researchers are very familiar with. From simple tricks for laboratory work to choosing the right software or planning the next career steps, this was a reoccurring theme during the career of the author, who will try to give a short personal overview for young researchers, especially in the analytics and/or natural products field. These topics and lists represent a personal opinion and are neither meant to be all‐encompassing nor of course might differ from the experiences of other researchers.

1

As a rather recent PhD graduate and still an “early career researcher”, the author wondered what to write about that would be interesting for a young scientist. The answer came while overhearing students in the break room stating, “I wish I had known all that before starting my PhD that would have made everything easier!” – An experience many researchers are very familiar with. From simple tricks for laboratory work to choosing the right software or planning the next career steps, this was a reoccurring theme during the career of the author, who will try to give a short personal overview for young researchers, especially in the analytics and/or natural products field. These topics and lists represent a personal opinion and are neither meant to be all‐encompassing nor of course might differ from the experiences of other researchers.

## 2 “SHOULD I DO A PHD?

Most more experienced readers likely made that decision long ago, but it is also the most frequently asked question to come across from graduating students. This decision is not only very important but also very personal. Ideally, you should make general plans about your future career early on and base your decision on that. As general advice, read job descriptions for jobs you are interested in and evaluate their requirements. Especially academic and research‐related industry jobs will often require a PhD, whereas jobs in fields like sales or marketing may have different requirements. A PhD is also not always easy and can take a lot out of young researchers, but likewise represents the chance to become fascinated with a topic, gain in‐depth knowledge and be a truly inspiring time. As recent studies have shed light on the mental health crisis in many PhD students,^1^, ^2^, ^3^ the author can only highlight the importance of keeping a work‐life balance, even in times of high pressure. Start by talking to your supervisor and/or colleagues if you feel completely overwhelmed and ask for help restructuring your work and finding some balance. Also especially in stressful times, stick with your hobbies and sport to get some balance and distance from your work. Finally, you can see if your university offers help, for example, counselling or anti‐stress workshops to better deal with stressful times. No one benefits from a burned‐out researcher. Nevertheless, the author agrees with a statement in this recent study that a “PhD with passion is a one‐time experience”.^4^

## 3 “BE AWARE OF NEW TECHNOLOGIES AND SOFTWARE”

New technologies and method development lead to consistent improvement and implementation of new laboratory procedures. The analytical science field is thereby especially driven by the development of novel and improved equipment. In the field of, for example, chromatography and mass spectrometry (MS), large advances in the last decades, have not only implemented new techniques such as 3D high‐performance liquid chromatography (HPLC), modern ultra‐performance liquid chromatography systems or improved stationary phase materials to increase the separation efficiency and throughput of liquid chromatography but likewise saw a stark increase in the capability of mass spectrometers themselves. From MS‐based imaging techniques^5^ to utilizing ion mobility^6^ or the development of magnetic resonance mass spectrometers,^7^ new technologies consistently improve what can be achieved by (LC‐) MS equipment. Recent technologies further improved measurement speeds, by replacing the LC‐separation through, for example, an automated solid‐phase extraction (SPE) step (SPE‐MS)^8^ or acoustic droplet ejection^9^ to utilizing acoustic mist ionization MS^10^ for “ultra‐high throughput” MS.^11^ The increase of measurement speeds and the corresponding amount of data however also highlights the need for more streamlined and automated analytic workflows.

While young researchers will rarely have the chance to choose which instrument to acquire, it is always wise to stay up to date with recent developments and trends. Especially together with the increased implementation and affordability of laboratory automation, as liquid handling robots, HTP experiments in 96‐ or 384‐well plate formats, are becoming more frequent and enormous datasets come with it that require software‐based solutions.^12^, ^13^, ^14^ While most researchers often start using vendor‐specific software, the question arises on how to deal with larger or very specific datasets, for example, in the field of proteomics, metabolomics or MS imaging (MSI).

Luckily, there is not only a wide variety of commercial and free software available but also a growing analytics/bioinformatics community driving further improvements. The diversity and limitations however can make it hard, even for experienced researchers, to know which tools to choose or test out. Most analytical laboratories will already have their favourites and experienced colleagues to ask, but if you are one of the few users or start a different project, you might ask yourself “what do I choose/ where do I look”. There are many great reviews published highlighting the diversity of MS‐related software, for example, metabolomics,^15^, ^16^, ^17^ proteomics^18^, ^19^, ^20^ or MSI,^5^, ^21^, ^22^ that represent a good start. Besides that, a few aspects can help a novice user make a good choice:

### 3.1 Vendor‐specific or external software?

All MS devices from different vendors (e.g. Agilent, Bruker or Waters) comes with software to view your spectra and perform basic operations like creating an extracted ion chromatogram. They often offer additional (expensive) software for specific fields, for example, protein analysis or metabolomics. Positive hereby is an often‐well‐designed user interface and available support and training. On the other hand, most of these are specific for data recorded with one instrument, thus cannot analyze data stemming from “foreign” instruments, which can be critical if the research groups own diverse instrumentation. Most vendor‐independent software however is also independent of specific raw file formats and works with universal MS data formats such as mzML (or imzML for MSI), to which all raw data can be converted with free tools such as MS convert (Proteowizard Toolbox, Table 1). Have a look through the cited reviews and software to find suitable software to aid and speed up your data analysis. While the right choice of course depends on your research goals, a short exemplary selection of project‐specific software can be found in Tables 1, 2, 3, 4. Furthermore, the following advice was always useful to the author:

**TABLE 1:** Authors' selection of useful proteomics software

**Proteomics**					
**Name**	**Developer**	**Comment**	**Format**	**Availability**	**Link**
Skyline^23^	MacCoss Lab Software	Analysis of SRM/ MRM/ PRM/ DIA experiments	Download	Free	https://skyline.ms
Protein prospector^24^	UC San Francisco (USA)	Online Proteomics Toolbox	Online	Free	https://prospector.ucsf.edu
Mascot server^25^	Matrix Science	Online Proteomics Toolbox	Online	Free/license	https://www.matrixscience.com
ProteoWizard^26^	ProteoWizard (Diverse)	Diverse framework for proteomics workflow	Download	Free	https://proteowizard.sourceforge.io
NIST Peptide library^27^	NIST	Peptide library	Download	Free	https://chemdata.nist.gov
Proteomatic^28^	University of Münster (Germany)	Script‐based (GUI available)	Download	Free	https://www.uni‐muenster.de/hippler/proteomatic/index.html
Trans Proteome Pipeline (TPP)^29^	Seattle Proteome centre (USA)	Large‐scale proteomics	Download	Free	http://ww.peptideatlas.org
ProteomeTools^30^	Diverse	Human Proteome spectral libraries	Download	Free	http://www.proteometools.org
Proteomics Toolkit	Institute for systems biology (Seattle, USA)	Different calculation tools, for example, fragment ions or enzymatic digestions of proteins	Online	Free	http://db.systemsbiology.net:8080/proteomicsToolkit

**TABLE 2:** Authors' selection of useful metabolomics software

**Metabolomics**					
**Name**	**Developers**	**Comment**	**Format**	**Availability**	**Link**
MS DIAL^31^	Dr Hiroshi Tsugawa (RIKEN Center for Sustainable Resource Science, Japan)	Software	Download	Free	http://prime.psc.riken.jp/compms/msdial/main.html
MS Finder^32^	Dr Hiroshi Tsugawa (RIKEN Center for Sustainable Resource Science, Japan)	Software	Download	Free	http://prime.psc.riken.jp/compms/msfinder/main.html
MZmine 3^33^, ^34^	Diverse	Software	Download	Free	http://mzmine.github.io/
GNPS^35^	Scripps (UCSD, USA)	Online tool and metabolite library	Online/download	Free	https://gnps.ucsd.edu/
MassBank^36^	Leibniz Institute of Plant Biochemistry/ Helmholtz Centre for Environmental Research / University of Luxembourg	Metabolite library	Online/download	Free	https://mona.fiehnlab.ucdavis.edu/
Metlin^37^, ^38^	Scripps (UCSD, USA)	Online tool and metabolite library	Online/download	Free	https://metlin.scripps.edu/
Metlin Gen2^37^, ^38^	Scripps (UCSD, USA)	Online tool and metabolite library	Online/download	License	https://massconsortium.com/
Metabo‐ Analyst^39^	Xia Lab, McGill University (Canada)	A broad set of tools for MS data processing, metabolomics and statistical analysis	Online	Free	https://www.metaboanalyst.ca/home.xhtml
Metabolights^40^, ^41^	EMBL‐EBI (European Bioinformatics Institute, UK)	Database for Metabolomics experiments and derived information	Online/download	Free	https://www.ebi.ac.uk/metabolights/index
MetaboScape	Bruker	All‐in‐one suite for metabolomics, lipidomics and phenomics	Download	License	https://www.bruker.com/
NIST Mass spectral library	National Institute of Standards and Technology (USA)	Metabolite library	Download	License	https://chemdata.nist.gov/
SIRIUS^42^	Böcker group (University of Jena, Germany)	Small molecule analysis	Download	Free	https://bio.informatik.uni‐jena.de/software/sirius/
MetEx^43^	Mo Lab (Princeton University, USA)	Online tool for HTP experiments	Online	Free	https://mo.princeton.edu/MetEx/
MetFrag^44^	deNBI (German Network for Bioinformatics Infrastructure)	Metabolite annotation against in‐silico‐generated spectra	Online/download	Free	https://ipb‐halle.github.io/MetFrag/
Galaxy^45^	Workflow4metabolomics	Metabolomics workflow and online community	Online/download	Free	https://workflow4metabolomics.org/galaxy‐environment

**TABLE 3:** Authors' selection of useful MS imaging (MSI) software

**Imaging (MSI)**					
**Name**	**Developers**	**Comment**	**Format**	**Availability**	**Link**
BASIS^46^	Computational and Systems Medicine (Imperial College London, UK)	Large‐scale MSI data processing	Download	Free	https://bitbucket.org/iAnalytica/basis_pyproc/src/master/
BioMap^47^	Novartis	4D representation (3 dimension and free to choose 4th dimension); requires IDL Virtual machine	Download	Free	https://ms‐imaging.org/mass‐spectrometry‐imaging‐society/
CreateTarget/Analyze This!^48^	University of Leuven (Belgium)	Enables MS imaging from Bruker data together with BioMap	Download	Free	http://maldi‐msi.org/
MSiReader^49^	Mathworks/Matlab	Requires Matlab	Download	Free	http://www.msireader.com/
SCiLS	Bruker	MSI data visualization and analysis, vendor‐independent	Download	License	https://www.bruker.com/
Datacube Explorer^47^	AMOLF (Amsterdam, Netherlands)	Visualization of (very) large MSI datasets	Download	Free	https://amolf.nl/download/datacubeexplorer
Spectral‐Analysis^50^	NiCE‐MSI (United Kingdom)	Processing and analysis of MSI data	Download	Free	https://github.com/AlanRace/SpectralAnalysis
msiQuant^51^	Uppsala University (Sweden)	Display and analysis of large MSI data sets, independent of the manufacturer (using imzML data format)	Download	Free	https://ms‐imaging.org/paquan/
OpenMSI^52^	Lawrence Berkeley National Lab (USA)	MSI data visualization and analysis	Online	Free (registration required)	https://openmsi.nersc.gov/openmsi/client/
METASPACE^53^	Alexandrov team (EMBL)	Metabolite annotation from MSI data	Online	Free	https://metaspace2020.eu/
rMSI‐annotation^54^	University Rovira (Spain)	Metabolite annotation from MSI data	Download	Free	https://github.com/prafols/rMSIproc
Mirion^55^	University Giessen (Germany)	Access by contacting developers	x	Free	bernhard.spengler@anorg.chemie.unigiessen.de
Lipostar MSI^56^	Molecular Horizon	MSI data visualization and analysis, Free trial and reduced academic licence	Download	License	https://www.molhorizon.it/software/lipostarmsi/

**TABLE 4:** Authors' selection of generally useful software that young researchers might benefit from

**Additionally useful**					
**Name**	**Developers**	**Comment**	**Format**	**Availability**	**Link**
OpenMS^57^	deNBI (German Network for Bioinformatics Infrastructure)	Viewing and analyzing general MS data and creating workflows	Download	Free	https://www.openms.de/
Mass++^58^	Shimadzu	Viewing and analyzing general MS data	Download	Free	https://bio.tools/mass
mMass^59^	Martin Strohalm	Viewing and analyzing general MS data, not developed anymore	Download	Free	http://www.mmass.org/
Mass Spec calculator	Quadtech Associates	Fragment prediction	Download	Free	https://www.quadtechassociates.com/
MS‐utils.org	Magnus Palmblad	Very large list of MS Software for different applications	Online	Free	https://ms‐utils.org/
Github	Github, Inc	Software development + repository	Online/download	Free	https://github.com/
R‐Studio	Rstudio, Inc	Software for datascience (using “R”)	Download	Free	https://www.rstudio.com/
Cytoscape^60^	Diverse	Software platform for visualizing complex networks, for example, from GNPS	Download	Free	https://cytoscape.org/
Antismash^61^, ^62^	Diverse	Genome mining platform	Online	Free	https://antismash.secondarymetabolites.org
Biorender	Biorender	Graphic software for scientific figures (with templates)	Online	Free/license	https://biorender.com/
Inkscape	Inkscape	Graphic software for vector graphics	Download	Free	https://inkscape.org/de/
Vectr	Vectr	Graphic software for vector graphics	Online	Free	https://vectr.com/

### 3.2 How old is the software, is it still being maintained/updated (e.g. on GitHub)?

Especially with non‐commercial software, there is no guarantee that it is further developed, or any support is available. As it can be very frustrating choosing software for your analysis workflow, just to discover that you cannot fix small bugs, always check if there is an active community using the software (and citing it) and if there is support available if you get stuck.

### 3.3 Does the software have a graphical user interface or is it based on scripts?

Even though most commercial scientific software has more‐or‐less intuitive graphical user interfaces (GUIs), to ease the use of the software, many very powerful non‐commercial tools are often based on using scripts written in coding languages, for example, R or Python. For users with no bioinformatics background, using software with a good GUI can reduce a lot of complexity at the beginning. However, from personal experience, the author can only recommend trying to get a basic understanding of these coding languages and trying out freely available scripts published in repositories such as GitHub. A lot of them are driven and consistently optimized by a large scientific community and offer the opportunity to tailor a workflow very specifically and ask about it.

Especially when students are just starting out, it is very helpful to have a few standard workflows and methods to follow. The more you get settled, it is always wise to question the “status quo” and see if you can improve workflows and methods, starting from your measurement parameters to the analysis software. In order to give a few examples and showcase the increasing diversity of specialized MS analysis software, readers can find a selection of different MS‐application in Tables 1, 2, 3, 4, and are further referred to the earlier cited reviews.

### 3.4 “Money is always tight – But there are tricks to safe it”

Even though the founding of research groups can differ massively, even within departments, money is always an issue. Everything, from scientific software and access to publications to single‐use equipment, is rather expensive, often leading to a hard decision on what to spend the limited resources on. There are however some good ways to save money and resources that might not be obvious to young researchers. As described above, there is a great diversity of free software and databases available, which are often able to replace expensive licensed variants. While, for example, in the metabolomics fields, software like MassHunter (Agilent) or MetaboScape (Bruker), are often the first choices depending on used instrumentation, freeware such as MS DIAL^31^ or MzMine^33^, ^34^ can likewise accomplish many of the offered functions of licensed software. Similarly, one has to evaluate if money should be allocated to purchase spectral metabolite databases, for example, Metlin Gen2^38^ or the NIST spectral database (Table 2) or if freely available and community‐driven databases such as GNPS^35^ or Massbank^36^ are already sufficient for one's approach.

Due to the increase in sample sizes and implementation of HTP workflows, the amount of single‐use plastic has recently become a focus for financial and ecological awareness also in academic settings.^63^, ^64^ There can be of course good arguments for single‐use equipment, for example, in GMP or medical settings, but in many situations, the reuse of disposable equipment is absolutely possible, even though discouraged by the vendors for obvious reasons. The author can only urge young researchers to think about their workflow, and used equipment and search for, or develop reuse protocols when possible and reasonable. There are already multiple protocols published to regenerate and reuse “disposable” equipment starting from cleaning reaction tubes to DNA‐purification columns,^65^, ^66^ protein filter units^67^, ^68^ up to SPE filter tips, columns or disks.^69^ The reuse of equipment of course carries the risk of sample contamination through carry‐over and should always be thoroughly investigated before being implemented. The author has personally tested the reuse, for example, diverse SPE material up to five times without detection of any cross‐contamination, which is in agreement with another study in which SPE material could be repurposed up to 10 times.^69^

### 3.5 Reach out to other researchers/collaborate

The author can only recommend connecting with other researchers in your field (and beyond) from a very early stage. Attend conferences if you can. There are many that specifically call on early career researchers to contribute and they are not only a great chance to practice presenting your own research but valuable to gain new inspirations and advice for your lab work and help build your own network of peers. A lot of conferences and organizations offer grants one can apply for to support the costs of conference participation and travel. Do not leave this up to your supervisor, be proactive, look for fitting conferences and grants and ask if you can present there and/or apply for funding. It is always helpful to gain some outside perspective and likewise see the newest trends and results in your field. Not only that but you might meet your next collaborator or future research group there. This also applies to publications. Most researchers will be happy to share their papers with you if you ask them and furthermore contact authors if you cannot replicate their published results. Most will be more than welcome to offer help or advice, but you need to reach out!

### 3.6 Invest in additional skills

Even though your PhD topic and the methods coming with it are likely overwhelming enough at the beginning, it is always wise to invest some time to learn additional (soft‐) skills. Most researchers will benefit from having a basic understanding of coding languages and presentation skills to explain their research to diverse audiences. Even though you are a scientist foremost, the author can only recommend the training to present your research and design good figures early on. Even the best results can fail to interest your peers if presented and explained badly. Besides using specific scientific software to create basic figures, it can be wise to use more professional graphics software. While some departments might have licenses for professional software such as Adobe Illustrator or Corel Draw, there are again free alternatives available, for example, Vectr (Table 4). For scientific schemes, specific software such as Biorender, providing diverse templates, can additionally save a lot of time and yield better designs. Finally, do not underestimate the importance of interpersonal skills. Teaching experiences are not only sought after in academic settings but are pretty much essential for any future leadership roles, in which you have to supervise students or employees. In the same regard, invest in project management and organizational skills to keep your work on track.

In general, obtain useful additional skills, even if they are not essential for your current research. No matter if you want to stay in academia or find a position in the industry, think about your CV and the skills you have and might need in the future. If you have a basic idea of where you want to end up, look up these positions and what requirements they have early and try to learn some of them when possible. Especially if you want to transfer into the industry, make sure your skills are needed there or even with a large publication output you will have limited chances to get the position you want.

### 3.7 A few last words

The author of this text, like most (former) PhD students, has experienced great and exciting, as well as very frustrating days or weeks where nothing worked the way, it was supposed to. While the latter can be especially demotivating, learning from mistakes and testing different approaches is as much a part of the learning process as finally getting those publishable results. This however does not mean that you cannot learn from the experiences of others and avoid the same frustration or mistakes. The author hopes that the advice given here will motivate young researchers to make use of state‐of‐the‐art techniques and software, safe resources where possible and maybe even find new approaches to make your PhD life a little easier and more exciting.

## CONFLICT OF INTEREST

The author declares no conflict of interest.
